# Evaluating Neurogenic Bladder Management in Palestinian Healthcare: A Qualitative Study

**DOI:** 10.7759/cureus.64799

**Published:** 2024-07-18

**Authors:** Mothana Sawafta, Mohammad Abushamma, Khaled Jallad

**Affiliations:** 1 Emergency Center, Jenin Governmental Hospital, Jenin, PSE; 2 Obstetrics and Gynaecology, Palestinian Medical Complex, Ramallah, PSE; 3 Primary Health Care, Primary Health Care Corporation, Tulkarm, PSE

**Keywords:** life consequences, health sequelae, spinal cord injuries, spina bifida, multiple sclerosis, lower urinary tract dysfunction, quality of life, urologic conditions, neurogenic bladder

## Abstract

Background: Neurogenic bladder (NB) is a prevalent urologic condition significantly impacting the health and quality of life of affected individuals. The condition, often resulting from various etiologies such as spinal cord injuries and multiple sclerosis, leads to severe life problems, including pain and impaired physical, mental, social, and emotional functioning. This study aims to explore the medical practices of urologists in the diagnosis, management, and care of NB patients within the Palestinian healthcare context, highlighting the absence of a unified treatment protocol and the reliance on private clinics for care.

Methods: An exploratory qualitative study design was employed, adhering to the Consolidated Criteria for Reporting Qualitative Research (COREQ) checklist. Structured interviews were conducted with 14 urologists and two urology residents across eight different cities in Palestine, including 10 governmental hospitals, two private hospitals, one university hospital, and one charity hospital. Fourteen doctors had private outpatient clinics alongside their work in hospitals. A questionnaire developed by the authors was delivered to specialists and residents to understand the evaluation, management, follow-up practices, and challenges faced in treating NB patients. The study focused on the diagnostic processes, treatment modalities, complications management, and the impact of the lack of standardized protocols on patient care.

Our qualitative study consists of six major themes, each theme consisting of multiple sub-themes and different participant responses: (1) diagnosis and follow-up of NB patients; (2) general issues in the management of NB; (3) evaluation and follow-up of upper and lower urinary system function in NB patients; (4) urinary tract infections associated with NB disease and how to deal with it; (5) opinions and future attitudes in the treatment of NB patients; (6) NB in patients with multiple sclerosis.

Results: The study found that urodynamic studies are crucial in NB diagnosis, yet there is no unified management protocol, leading to varied practices. Most participants preferred the American Urological Association (AUA) guidelines in the absence of Palestinian protocols. Six major themes emerged, including diagnosis and follow-up challenges, general issues in NB management, evaluation and follow-up of urinary system function, urinary tract infections management, opinions on future treatment directions, and specific considerations for NB patients with multiple sclerosis.

Conclusions: The study highlights the need for a unified, standardized protocol for the management of NB patients in Palestine. The reliance on international guidelines, primarily the AUA protocols, underscores the gap in local healthcare policies. The findings call for the establishment of national guidelines and enhanced resources for the effective management of NB, aiming to improve patient outcomes and quality of life.

## Introduction

Neurogenic bladder (NB), a common urologic condition, significantly impacts the health and quality of life, characterized by dysfunction in urine storage and elimination due to neurological damage from conditions such as spinal cord injuries, multiple sclerosis, and Parkinson's disease [1-3]. Common symptoms include urinary incontinence, urgency, frequency, and difficulty in emptying the bladder, often leading to challenges related to urinary tract infections (UTIs) [4]. These symptoms, associated with pain, worsen the physical, mental, social, and emotional functioning of patients [1]. So this highlights the importance of tailored management strategies to enhance their quality of life [3,4].

Delgado et al. conducted a review exploring the management of UTIs in adult patients with neurogenic lower urinary tract dysfunction, emphasizing the complexity and evolution of this field [5]. Non-surgical strategies, such as antibiotic therapies and bladder instillations, are typically the first line of treatment, with surgical intervention considered carefully as a last resort or based on patient and caregiver preference [5]. In another study, Nevedal et al. investigated the experiences of women with spinal cord injuries living with NB [6]. In this qualitative study involving 50 women, bladder symptoms were spontaneously mentioned by the majority of participants, with six major themes emerging from the interviews: life controlled by bladder symptoms, urinary incontinence, challenges relevant to women's issues, medical management, and seeking independence.

Recently, the lower urinary tract symptoms treatment constraints assessment (LUTS-TCA) was validated as a tool to evaluate NB treatments, demonstrating excellent psychometric properties [1].

NB management varies worldwide, which reflects the same situation in Palestine, particularly between resource-rich and resource-poor regions, with a pressing need for global collaboration to improve care and outcomes [7]. Public health strategies should prioritize the development of treatment guidelines, promote best practices, and include both pharmacological and surgical options to enhance patient care [8]. Economic studies highlight clean intermittent catheterization as a cost-effective management strategy, emphasizing the integration of cost and clinical effectiveness in public health policy for NB [9]. Holistic approaches must incorporate guideline development, interdisciplinary collaboration, and financial evaluations to ensure patient-centered, efficient, and effective NB treatment.

Previous studies have examined new treatments for NB, evaluating their success and impact on patient health and function. Through clinical trials and feedback from patients, these efforts aim to enhance NB care. The study provides important findings on the effectiveness of these treatments, aiming to better the lives of individuals dealing with this difficult condition. Gharbi et al. reviewed the quality of life in neurogenic patients based on different bladder management methods, concluding that intermittent catheterization generally offers a better quality of life due to fewer infections and greater independence, despite the need for adherence to a strict schedule [10]. Previous studies also revealed that individuals with gradual, non-traumatic neurological conditions face a significantly reduced risk of damage to the upper urinary tract and kidney failure, in contrast to those affected by spinal cord injuries or spina bifida. This variance in health outcomes is vital for tailoring treatment plans for NB issues [11].

This study addresses the diagnosis and management of NB in Palestine, focusing on where and how patients are diagnosed. The primary objective is to evaluate the adequacy and effectiveness of current protocols used by specialists and residents in treating this condition. The secondary objectives include assessing whether these protocols, particularly those provided by the Palestinian Ministry of Health, are sufficient based on clinical experience, identifying complications such as noncompliance with medications, side effects, drug interactions, and psychological care, and examining the management of infections associated with NB. Additionally, the study aims to explore future treatment expectations for NB in Palestine. Most NB patients in Palestine receive care in private outpatient clinics, with fewer being treated in specialized centers and hospitals, leading to a significant portion of patients not receiving adequate management. The study aims to understand the treatment protocols in use, their effectiveness, and the challenges faced in managing this condition.

## Materials and methods

Study design

This study is being reported in adherence to the Consolidated Criteria for Reporting Qualitative Research (COREQ) checklist [12]. In this exploratory qualitative study, we recruited and conducted structured interviews with urologists and residents of the urology department. These interviews were conducted in clinics and over voice calls to explore how NB patients are being evaluated, managed, and followed up, what are the complications that these patients face and how these complications are dealt with, and, lastly, what they hoped to be improved in the management of this condition. These interviews were conducted from 10 March 2021 to 24 March 2021 and 14 March 2024 to 27 March 2024.

Sampling and determination of the sample size

The sampling was based on the judgmental method, which was used to recruit and invite interviewees for this study. The interviewees were identified through personal contacts in the discipline. Potential interviewees were approached, invited, and recruited based on their prior knowledge and experience in dealing with NB patients [13]. A total of 16 specialists and residents from eight different cities in Palestine were distributed across 10 governmental hospitals, two private hospitals, one university hospital, and one charity hospital, which were included in the study (Appendix 1) [14]. Fourteen of those who were recruited were urologists with more than five years of experience, and two of them were residents of the urology department. Fourteen doctors had private outpatient clinics alongside their work in hospitals. A questionnaire developed by the authors was delivered to specialists and residents. The interviewees gave informed consent before they were interviewed, and the interviews were audibly recorded. In this study, the thematic saturation point was used to approximate the sample size that was imperative. The recruitment endpoint was determined depending on having sufficient and different responses for the themes and sub-themes [12].

Interview schedule

The interview schedule used in this study was obtained from past conduct of qualitative studies and questionnaires. The questions covered in the interviews were related to diagnosis of NB, caring for these patients, treatment, evaluation, and follow-up of lower and upper urinary tracts, dealing with UTIs associated with NB, opinions and future directions in the treatment of NB patients, and NB in patients with multiple sclerosis [15].

Data collection

The interviews were conducted by Dr. Hatim Hijaz and graduating medical students who had experience in conducting qualitative studies. The interviewer was a urologist at An-Najah National University Hospital at the time of the study. Structured interviews were conducted using a guide that contained a set of questions, which are classified into thematic and sub-thematic. All interviews were audio-recorded and transcribed verbatim and some of them were done by face-to-face interview due to the COVID-19 pandemic before analysis. Doctors' answers were approved after transcripts were completed. Interviews were not repeated.

The transcripts were carefully reviewed, and the data were coded to identify recurring themes and sub-themes. The analysis focused on six major themes: (1) diagnosis and follow-up of NB patients; (2) general issues in the management of NB; (3) evaluation and follow-up of upper and lower urinary system function; (4) management of urinary tract infections associated with NB; (5) opinions and future directions in treatment; and (6) management of NB in patients with multiple sclerosis.

Each theme was further divided into sub-themes, which provided a detailed understanding of various aspects of NB management. The thematic analysis was conducted iteratively, with the researchers refining the themes and sub-themes through continuous comparison of the data. This method ensured that the analysis captured the complexity of the participants’ experiences and perspectives, providing a comprehensive overview of NB management in the Palestinian context.

Ethical approval

The Institutional Review Board (IRB) of An-Najah National University, Nablus, approved this study. Interviewees provided audio-recorded informed consent before the interviews began. The recordings were transcribed verbatim into a Microsoft Excel (Microsoft Corporation, Redmond, WA) document.

## Results

Interviews and response rate

We invited 20 urologists and residents of the urology department from different hospitals to participate in the interview. Sixteen (80%) were willing to participate in the study and four (20%) declined for unknown reasons.

Sociodemographic and practice characteristics of the interviewees

The interviewees were all males, including 14 urologists and two residents in the urology department. Details of the socio-demographic and practice characteristics of the interviewees are shown in Table 1. The median duration of the interview was around 30 minutes.

**Table 1:** Sociodemographic and practice characteristics of the interviewees.

Characteristics	N = 16	%
Gender		
Male	16	100
Female	0	0
Age (years)		
<35	5	31.25
=35	3	18.75
>35	8	50
Year of graduation		
<2010	10	62.5
>2010	6	37.5
Total patients treated per month		
<50	7	43.75
>50	9	56.25
Total neurogenic bladder patients treated per month		
<5	7	43.75
=5	2	12.5
>5	7	43.75
Years of experience		
<5	2	12.5
>5	14	87.5
Presence of the urology department		
Yes	8	50
No	8	50
Presence of urodynamic study machine		
Yes	4	25
No	12	75
Hospital type		
Governmental	10	62.5
University	3	18.75
Private	2	12.5
Charity university	1	6.25

Quality indicators: themes, sub-themes, and patterns

Details of these themes and sub-themes are shown in Table 2. Illustrative quotes that support the themes and sub-themes are presented in Tables 3-8.

**Table 2:** Themes and sub-themes in the management of neurogenic bladder patients.

Themes	Sub-themes
Diagnosis and follow-up of neurogenic bladder patients	Diagnosis process
	Protocols and guidelines
	Care and follow-up
	Complications
	Systemic review
General issues in the management of neurogenic bladder	Bladder emptying
	Treatment steps
	Medication management
	Psychological health
Evaluation and follow-up of upper and lower urinary system function in patients with neurogenic bladder	Upper urinary tract
	Lower urinary tract
Urinary tract infections associated with neurogenic bladder disease and how to deal with it	Urine culture
	Asymptomatic bacteriuria
	Symptomatic UTI
	Antibiotic prevention
	Treatment protocols
Opinions and future attitudes in the treatment of neurogenic bladder patients	Current care and treatment
	Future recommendations
Neurogenic bladder in patients with multiple sclerosis	Additional tests
	Diagnosis
	Unified protocols

**Table 3:** Illustrative quotes for diagnosis and follow-up of neurogenic bladder patients.

Box 1: Illustrative quotes for diagnosis and follow-up of neurogenic bladder patients	
Diagnosis process	1. “These patients are usually diagnosed in outpatient clinics, they are approached by taking patient history, physical examination, running some laboratory test like kidney function test, if I suspect neurogenic bladder, I perform pre and post-voiding ultrasound and then I refer them to centers with a urodynamic machine in order to confirm diagnosis" (EQ, Governmental). 2. "Usually, these patients tend to be visiting lots of private clinics without getting the correct diagnosis, but they are diagnosed at our hospital (An-Najah National University Hospital), due to the availability of specialized tools and machines to do so, we also do history physical examination, laboratory test, and ultrasound to see the renal pelvis" (AA, University). 3. "We mainly depend on the history and physical examination then we perform laboratory tests such as urinalysis, urine culture, prostate-specific antigen (PSA), kidney function test. Also, we do pre and post-voiding ultrasound and we can perform MRI for brain and spinal cord. But if urodynamic machine is present, we diagnose the neurogenic bladder depending on its results" (MBH, Charity University). 4. "Mostly these patients are discovered by chance, and they present to the clinic due to recurrent UTIs, incontinence, and by doing cystourethrogram we can discover neurogenic bladder, and a good percentage of our patients are considered chronic cases because they don't visit doctors until it’s affecting their daily life" (HY, Private).
Protocols and guidelines	1. "In our hospital, we depend on the American and British protocols for the diagnosis, but there is no specific Palestinian protocol " (AMA, Private). 2. "There is no specific protocol for diagnosis and doctors depend on experience" (HH, University). 3. "In our hospital (Tulkarm Governmental Hospital) there is no specific protocol or guidelines but we use AUA protocol" (EA, Governmental).
Care and follow-up	1. “After we diagnose, we give them the right management and we follow them up with post-voiding residual urine ultrasound and they asked to be routinely presenting to the clinic, in Ministry of Health, there are urologists but no urodynamic machine, we depend on the American and British protocols for follow up, but there is no specific Palestinian protocol" (AGA, University). 2. "No united protocol for the follow-up" (HY, Private). 3. "There are no special services in the centers of Ministry of Health and we take care of the patients through the family and regular follow-up in clinics, there is no particular protocol for follow up the patients" (AQ, Governmental).
Complications	1. "Patients may experience UTI, bilateral hydronephrosis, renal impairment, urine stasis, and stone formation" (NS, Governmental). 2. "The main complications are urinary incontinence, urine retention, and urine reflux" (AA, University). 3. "Patient might have renal impairment and recurrent UTI as a complication of neurogenic bladder” (MBH, Charity University).
Systemic review	1. “Yes, patients are asked about symptoms related to different body systems and spinal cord injury" (SAD, Governmental). 2. "Patients are systematically reviewed especially for spinal cord injuries or brain abnormalities" (AMA, Private). 3. "Yes, we ask the patients about symptoms related to other body systems mainly spinal cord problems to rule out spina bifida and spinal cord injury" (HH, University).

**Table 4:** Illustrative quotes for general issues in the management of neurogenic bladder.

Box 2: Illustrative quotes for general issues in the management of neurogenic bladder	
Bladder emptying	1. "We give patients appropriate treatment and put Foley’s catheter for them and we follow them up if there is improvement the catheter is removed but if not, we offer him Foley’s catheter of clean intermittent catheterization" (AA, University). 2. "If the reason for this is flaccid bladder or neurological defect, we use Foley's catheter or clean intermittent catheterization but if there is obstruction, we can do surgery" (MAM, Governmental). 3. "After I confirmed that the patient can't empty the bladder by examination and ultrasound, I ask the patient to strain to urinate, if can't, I will insert Foley's catheter then I do renal and post-voiding ultrasound" (MBH, Charity University).
Treatment steps	1. "We administer anticholinergic medication, and we do augmentation surgery" (HH, University). 2. "We can add alpha-blocker, also Botox injection or sphincterotomy may be used for the spastic neurogenic bladder. We can perform endoscopy with Botox injection or augmentation cystoplasty" (HY, Private). 3. "In case of overflow incontinence, we do clean intermittent catheterization every 8 hours and we add oxybutynin. We add Vesicare (solifenacin) for overactive bladder" (AZ, Governmental).
Medication management	1. "By showing the patients the complication in the form of videos about dialysis patients and how it’s difficult to do dialysis 3 times a week” (MS, Governmental). 2. "If we notice side effects of medication such as constipation, xerostomia, for constipation, we give laxatives and for xerostomia, we recommend to drink more fluids, if we didn’t manage to improve the side effects, we lower dose or change medication. We stop neurogenic bladder medication if the other medications are crucial, till we find a better medication" (HH, University). 3. "By explaining the consequences and complications of not complying to the medication such as renal failure and by doing routine visits to the clinic, we explain anticholinergic side effects to the patient, we avoid giving patients with chronic constipation anticholinergics, in case of severe side effects we can replace it we other types of medication. Drug interactions are common so we try to avoid these situations by drug modification, dose adjustment, and by strict follow-up" (AMA, Private). 4. "We always explain to the patients that they must adhere to the treatment and if not, they will suffer from irreversible renal failure. Medications are usually stopped if they cause severe problems, and in the case of persistent UTI due to catheter we do diversion of urine and may insert a suprapubic catheter. I change the neurogenic bladder medication regarding to priority of other medication that they take" (MBH, Charity University).
Psychological health	1. “We don’t refer them to a psychiatrist but we provide them social support" (MO, Governmental). 2. "Most neurogenic bladder patients have psychological issues, we reassure and explain the prognosis of the disease, but in some cases, we inform them by the need for psychosocial support“ (HH, University). 3. "Most of these patients have psychological problems, and they don't accept current conditions. Unfortunately, ashamed to ask them to visit a psychologist" (HY, Private).

**Table 5:** Illustrative quotes for evaluation and follow-up of upper and lower urinary system function in patients with neurogenic bladder.

Box 3: Illustrative quotes for evaluation and follow-up of upper and lower urinary system function in patients with neurogenic bladder.	
Upper urinary tract	1. "By doing ultrasound, nuclear imaging for renal function and kidney function test, we repeat urodynamic 6 months later and 1 week for the kidney function tests" (YH, Governmental). 2. "We follow them up by history physical examination, we do urine analysis, ultrasound and we can do further imaging like Demsa scan or MAG3 if there is obstruction and lastly urethroscopy, we follow up depends on patient condition if its acute or chronic condition, patient with renal impairment we repeat tests every 10-14 days" (HH, University). 3. "I follow them up with ultrasound, I repeat tests 6 months later" (MBH, Charity University).
Lower urinary tract	1. "By urodynamic study, post-voiding ultrasound, we repeat urodynamic 6 months later and 1 week for the ultrasound" (AGA, University). 2. "By doing urine analysis, kidney function test, cystoscopy, urodynamic study, we repeat them every 3-6 months" (AQ, Governmental). 3. "By doing kidney function test, ultrasound, and urine culture every 3 months" (AMA, Private).

**Table 6:** Illustrative quotes for urinary tract infections associated with neurogenic bladder disease and how to deal with it.

Box 4: Illustrative quotes for urinary tract infections associated with neurogenic bladder disease and how to deal with it	
Urine culture	1. “Urine culture is not routinely done unless there are specific urinary tract infection symptoms based on patient history” (AA, University). 2. "If patients had asymptomatic bacteriuria, we repeat culture after a month" (MBH, Charity University). 3. "Every 3 months" (EA, Governmental).
Asymptomatic bacteriuria	1. “Antibiotics are not given for asymptomatic bacteriuria unless the patient is a known case of diabetes, immunocompromised, or pregnant woman. Give them nitrofurantoin or trimethoprim-sulfamethoxazole 35 days" (HH, University). 2. "Observation and reassurance with follow-up every month. Depending on culture, and we treat for 14 days in neurogenic patient" (HY, Private). 3. "I give him low doses for a long time prophylactic antibiotics. Low dose nitrofurantoin for 24 days" (EQ, Governmental).
Symptomatic UTI	1. "If a patient has UTI, I give him 7-10 days nitrofurantoin, 3-5 days sulfa drugs, if symptoms persist more than 7 days, I use ciprofloxacin or levofloxacin" (AGA, University). 2. “Antibiotic for 2 weeks and after 3 days from the last treatment day we do a urine culture" (MBH, Charity University). 3. "According to culture, 14 days treatment” (EA, Governmental).
Antibiotic prevention	1. "I recommend using antibiotics in cases of recurrent UTI but not self-administered antibiotics in order to avoid antibiotic-resistant bacteria" (AA, University). 2. “No, we use antibiotics in neurogenic bladder patients depending on culture results, for the self-administered we just allow it for young sexually active females” (AMA, Private). 3. "I'm against this, if it was documented that the patients have recurrent UTI I give low dose long time prophylactic nitrofurantoin with antiseptic like outside or cranberry and alkalinization of urine" (NS, Governmental).
Treatment protocols	1. "There is no specific protocol, but we give prophylactic antibiotics, we focus on behavioral modification for these patients like increasing water intake and bladder rehabilitation by training the patients to micturate every 3-4 hours” (HH, University).

**Table 7:** Illustrative quotes for opinions and future attitudes in the treatment of neurogenic bladder patients.

Box 5: Illustrative quotes for opinions and future attitudes in the treatment of neurogenic bladder patients	
Current care and treatment	1. "In our hospital (AN-NUH), patients receive adequate management and we don’t need to refer patients to other centers" (AA, University). 2. "No, they don’t receive the optimal treatment and they usually refer to more specialized centers which can also provide rehabilitation" (SAD, Governmental).
Future recommendations	1. "They do not take their right because they need advanced tests for diagnosis like urodynamics and some patients need surgical treatment like augmentation cystoplasty" (MAM, Governmental). 2. "I’m looking for devices that help the bladder to contract if they have flaccid bladder" (HY, Private). 3. "I recommend founding new united protocols for the diagnosis and follow up of neurogenic bladder patients at a national level" (MBH, Charity University). 4. "Most neurogenic bladder patients need a urologist to follow them up, these patients should receive financial facilitation including the cost of diagnosis treatment and surgery, they should provide a sub-specialty in neurogenic bladder treatment" (HH, University).

**Table 8:** Illustrative quotes for neurogenic bladder in patients with multiple sclerosis (MS).

Box 6: Illustrative quotes for neurogenic bladder in patients with multiple sclerosis	
Additional tests	1. "Sometimes MRI ordered and consultation done by neurologist" (AGA, University). 2. "Usually, patients are dealt with and treated as neurogenic bladder and if no improvement after management we have to rule out other defects such as neurological diseases, also we can refer them to a neurologist" (AZ, Governmental).
Diagnosis	1. "About half of the patients that visit me have spinal cord injuries and discs, but MS patients are only two. MS patients are treated but never followed up" (MS, Governmental). 2. "Worldwide 33.33% of patients who have MS present with urinary symptoms, from personal experience, I’ve seen only 3 patients had MS" (HH, University). 3. "Yes, small percent but there is no difference in dealing with them in terms of treatment. I am following up 5-7 patients" (MBH, Charity University).
Unified protocols	1. "There is no united protocol for the urologists to deal with multiple sclerosis" (AA, University). 2. ”There is no united protocol for the urologists to deal with multiple sclerosis" (MO, Governmental). 3. "We treat neurogenic bladder condition indifferently whether it’s a primary or secondary, no united protocols in Palestine" (AMA, Private).

Diagnosis and follow-up of neurogenic bladder patients

Diagnosis Process

NB patients are usually diagnosed in outpatient clinics where the diagnosis process involves taking the patient's history, conducting a physical examination, and running laboratory tests like kidney function tests (Table 3). Pre- and post-voiding ultrasounds are performed, and if NB is suspected, patients are referred to centers with urodynamic machines to confirm the diagnosis. This process is often necessary because patients initially visit multiple private clinics without receiving an accurate diagnosis. Hospitals with specialized tools, such as An-Najah National University Hospital, provide a more thorough diagnostic approach.

Protocols and Guidelines

Hospitals typically rely on American and British protocols for diagnosis due to the absence of specific Palestinian guidelines. In the absence of unified protocols, doctors often depend on their experience and available international protocols.

Care and Follow-Up

After diagnosis, appropriate management is provided, and follow-up involves post-voiding residual urine ultrasounds and routine clinic visits. The lack of urodynamic machines in the Ministry of Health means follow-up care relies heavily on American and British protocols. There are no unified follow-up protocols, and care often depends on family support and regular clinic visits.

Complications

Complications from NB can include UTIs, bilateral hydronephrosis, renal impairment, urine stasis, and stone formation. Other complications noted are urinary incontinence, urine retention, and urine reflux.

Systemic Review

Patients undergo systematic reviews for symptoms related to different body systems, particularly spinal cord injuries or brain abnormalities, to rule out conditions like spina bifida.

General issues in the management of neurogenic bladder

Bladder Emptying

Treatment for bladder emptying in patients with NB includes the use of a Foley catheter or clean intermittent catheterization (Table 4). Surgery is considered if there is an obstruction. This approach is necessary when patients cannot empty their bladder despite efforts to strain during urination.

Treatment Steps

Management involves the administration of anticholinergic medications, alpha-blockers, Botox injections, and sphincterotomy. Augmentation cystoplasty is performed in severe cases. Clean intermittent catheterization is often used in cases of overflow incontinence, supplemented with medications like oxybutynin and solifenacin for overactive bladder.

Medication Management

Patients are educated about the complications of non-compliance through visual aids and detailed explanations. Side effects like constipation and xerostomia are managed with appropriate remedies, and medications are adjusted or replaced as necessary. Drug interactions are avoided through careful monitoring and adjustments.

Psychological Health

Psychological support is provided, though referrals to psychiatrists are not common. Patients are reassured about their prognosis, and in some cases, psychosocial support is recommended to help them cope with their condition.

Evaluation and follow-up of upper and lower urinary system function in patients with neurogenic bladder

Upper Urinary Tract

Follow-up for the upper urinary tract involves ultrasounds, nuclear imaging for renal function, kidney function tests, and repeat urodynamic studies depending on the patient's condition (Table 5). These evaluations are conducted regularly to monitor for potential complications.

Lower Urinary Tract

For the lower urinary tract, follow-up includes urodynamic studies, post-voiding ultrasounds, and repeated tests every few months to assess bladder function and detect any abnormalities.

Urinary tract infections associated with neurogenic bladder disease and how to deal with it

Urine Culture

A urine culture is performed based on specific UTI symptoms, with routine follow-up cultures conducted if necessary (Table 6). Cultures are repeated in cases of asymptomatic bacteriuria, especially in patients with diabetes, who are immunocompromised, or pregnant.

Asymptomatic Bacteriuria

Antibiotics are not given for asymptomatic bacteriuria unless the patient falls into a high-risk category. Long-term prophylactic antibiotics may be administered for prevention in these cases.

Symptomatic UTI

Treatment for symptomatic UTIs varies from seven to 14 days with antibiotics based on culture results. Follow-up cultures are done to ensure the infection has been eradicated.

Antibiotic Prevention

Prophylactic antibiotics are recommended for recurrent UTIs, focusing on behavioral modifications to prevent infections. Patients are advised against self-administering antibiotics to avoid resistance.

Treatment Protocols

There is no specific treatment protocol, but prophylactic antibiotics and behavioral modifications such as increased water intake and bladder rehabilitation are emphasized. Patients are trained to micturate every three to four hours to help manage their condition.

Opinions and future attitudes in the treatment of neurogenic bladder patients

Current Care and Treatment

Patients receive adequate management at hospitals like An-Najah National University Hospital, though specialized centers are often required for optimal treatment and rehabilitation (Table 7).

Future Recommendations

Recommendations include the need for advanced diagnostic tests like urodynamics and surgical treatments. Establishing national protocols and providing financial facilitation for patients are also suggested to improve care and management.

Neurogenic bladder in patients with multiple sclerosis

Additional Tests

MRI and consultations with neurologists are sometimes necessary for patients with suspected NB to rule out other neurological defects (Table 8).

Diagnosis

A small percentage of multiple sclerosis (MS) patients present with urinary symptoms, and they are managed similarly to other NB patients. There is no difference in treatment approaches based on whether the NB is primary or secondary to MS.

Unified Protocols

There are no unified protocols for managing NB in MS patients in Palestine. The treatment is based on general guidelines used for all NB conditions.

## Discussion

Summary of the main findings of the study

This qualitative study aimed to explore the practices and challenges faced by urologists in the diagnosis, management, and care of NB patients within the Palestinian healthcare context. The findings highlight significant gaps in the standardization of care and underscore the necessity for a unified treatment protocol tailored to the local healthcare system.

The study revealed that the diagnosis of NB in Palestine predominantly occurs in outpatient clinics through a combination of patient history, physical examination, and basic laboratory tests. Confirmatory diagnosis often requires referral to centers with urodynamic machines, a resource not uniformly available across all healthcare facilities. The absence of a unified national protocol results in varied diagnostic practices, with reliance on international guidelines such as those from the American Urological Association (AUA). This lack of standardization could lead to inconsistent patient outcomes and highlight an area needing urgent attention.

Management practices for NB in Palestine vary significantly due to the absence of localized treatment guidelines. Physicians frequently depend on international protocols or their own clinical experience. The primary management strategies include the use of anticholinergic medications, clean intermittent catheterization, and in severe cases, surgical interventions like augmentation cystoplasty. Medication management is complicated by the need to balance efficacy with the management of side effects and potential drug interactions. Additionally, there is a notable lack of structured psychological support for NB patients, which is crucial given the condition’s impact on mental health.

The follow-up care for NB patients involves regular monitoring of both upper and lower urinary tract function through ultrasounds, nuclear imaging, and repeated urodynamic studies. However, the frequency and type of follow-up assessments vary widely among practitioners. This inconsistency underscores the need for a standardized follow-up protocol to ensure comprehensive and consistent patient monitoring.

UTIs are a common complication in NB patients, and their management also suffers from a lack of standardization. The study found varying practices in the treatment of symptomatic UTIs and the use of antibiotics for asymptomatic bacteriuria. Prophylactic antibiotic use and behavioral modifications, such as increased water intake and bladder training, are recommended, but the absence of a standardized treatment protocol can lead to suboptimal patient care and increased antibiotic resistance.

The study participants expressed a strong need for the development of national guidelines and the establishment of specialized centers for NB management. The current reliance on private clinics and international protocols is insufficient to meet the needs of NB patients comprehensively. Recommendations for future improvements include the provision of advanced diagnostic tools, the development of unified treatment protocols, financial support for NB patients, and increased training for healthcare providers in the management of NB.

The management of NB in MS patients is particularly challenging due to the lack of specific protocols. While the treatment approaches for NB are generally applied to MS patients, the absence of tailored guidelines can result in inadequate care. The study highlights the need for protocols that address the unique needs of MS patients with NB to ensure they receive appropriate and effective treatment.

Integration of recent research findings

Recent studies offer a broader perspective on the management and treatment of NB, underscoring the importance of integrating both pharmacological and non-pharmacological approaches. For instance, a systematic review highlighted the moderate efficacy of treatments such as anticholinergics, mirabegron, and botulinum toxin, emphasizing the tailored treatment approaches for patients with neurogenic overactive bladder based on specific neurological conditions [16]. Another study focused on the physiological changes and management strategies for NB following spinal cord injury, advocating for risk stratification and interventions like botulinum toxin A for treating neurogenic lower urinary tract dysfunction [17].

There is a clear alignment between the practices of the interviewees and some of the key recommendations from recent research, suggesting a partial implementation of these findings. The study highlights the need for standardized protocols, which may further facilitate the integration of recent research into clinical practice.

Appraisal of the methods used in this study

In this qualitative study, urologists who were dealing with NB patients were interviewed in a set of structured interviews, which were conducted using a guide containing a set of questions. This is the first investigation in Palestine to explore the physician's role in the diagnosis of NB, caring for these patients, treatment options, evaluation and follow-up of lower and upper urinary tracts, dealing with UTIs associated with NB, opinions, and future directions in the treatment of NB patients and NB in patients with multiple sclerosis.

In this study, the interviewees were diversified to include urologists and residents in the urology department, who belong to different age groups, with variable years of graduation, have variable numbers of total patients treated per month and total NB patients treated per month, have different years of experience, and some of them work in urology department but the others do not. In some workplaces, a urodynamic study machine was present (Table 1).

Advances in treatment and management

The integration of findings from recent research underscores significant advances in the treatment and management of NB. The efficacy of treatments like botulinum toxin and mirabegron, as demonstrated in recent studies, supports the interviewed physicians' inclination toward adopting guidelines that incorporate a broad range of therapeutic options [18]. The emphasis on risk stratification and personalized treatment plans resonates with the need for a tailored approach to managing NB, which could be beneficial for the development of a Palestinian protocol [17].

Strengths and limitations of the study

The findings of this study might be interpreted after taking into consideration the following strengths and limitations. First, this study was a qualitative study in which stakeholders were interviewed. Adding a quantitative dimension could have further strengthened our findings. Second, all interviewees were from Palestine. This might further limit the generalizability of the findings. Third, the number of interviewees was relatively limited. The number of interviewees needed for this study was determined by the thematic saturation point. The interviewees were of one gender, had different academic backgrounds, were employed in different hospitals, and had long experience in the field. This might have added validity to the findings obtained in this study. Finally, data analysis was based on the interpretive description methodology. This method is associated with many challenges, such as limited resources, limitation of using a less-known method, and lesser certainty concerning the degree of interpretation sought.

The absence of a localized treatment guideline in Palestine is a critical gap identified in our study. The integration of recent research findings reinforces the importance of standardized treatment guidelines that are adaptable to the local healthcare context. Such guidelines should incorporate the latest evidence-based treatments and management strategies, including the use of botulinum toxin, neuromodulation, and the importance of psychological counseling in rehabilitation treatment [11,19].

Future directions

To enhance our research on NB management in Palestine, we plan to expand our study by increasing the sample size of participating urologists and residents and incorporating quantitative analysis. This will involve developing and deploying structured questionnaires to gather and statistically analyze data on diagnostic practices, treatment modalities, and follow-up procedures. Additionally, we will identify and explore areas of uncertainty such as the lack of standardized protocols, variability in diagnostic and treatment practices, and the need for psychological support. We will also evaluate emerging treatments and technologies, and their economic and policy implications. By adopting a mixed-methods approach and conducting longitudinal studies, we aim to develop comprehensive national guidelines tailored to the Palestinian healthcare context, ultimately improving patient outcomes and quality of life. There is also a need for further research on the efficacy and safety of emerging treatments, such as heat-sensitive moxibustion and engineered stem cells, in the context of NB after spinal cord injury [8,20].

## Conclusions

This study underscores the critical need for a unified and standardized protocol for managing NB in Palestine. The current reliance on international guidelines, such as those from the American Urological Association, highlights a significant gap in local healthcare policies. The absence of national guidelines results in varied practices among healthcare providers, leading to inconsistent patient outcomes. The findings emphasize the importance of developing national guidelines and enhancing resources to ensure comprehensive and effective management of NB. Addressing these issues is essential for improving patient care and quality of life for individuals suffering from NB in Palestine. Future efforts should focus on establishing dedicated centers for NB management, providing advanced diagnostic tools, and fostering collaboration among healthcare professionals to create a cohesive approach to treatment and follow-up care.

## Appendices

Appendix 1: Questionnaire

Theme 1: Diagnosis and Follow-Up of Neurogenic Bladder Patients

A. How to diagnose neurogenic bladder in Palestine? Where do they usually get diagnosed (private clinics/specialized centers)? What are the steps that are followed to diagnose neurogenic bladder?

B. What is the nature of the protocols and guidelines used in diagnosing neurogenic bladder disease? Are there protocols for practice in Palestine? Or does every doctor follow certain international protocols (American or European)?

C. How is the care of neurogenic bladder patients in Palestine? Are there dedicated services through the health facilities of the Ministry of Health? Or is the patient being followed up by doctors in the private sector? Are there special protocols followed to care for patients with neurogenic bladder in Palestine? Or does every doctor follow certain international protocols (American or European)?

D. What are the complications that usually occur in patients with neurogenic bladder who are followed up by you?

E. Are the patients asked about the symptoms of problems related to other body systems (systemic review), especially the nervous system?

Theme 2: General Issues in the Management of Neurogenic Bladder

A. What is your method to solve and treat the inability to empty the bladder?

B. High bladder filling pressure and urinary incontinence when treated only with clean intermittent catheterization - What is the next treatment step? High bladder filling pressure and urinary incontinence during treatment with clean intermittent catheterization and anticholinergic medication - What is the next step for treatment?

C. How do you deal with the side effects of medication and how do you deal with drug interactions?

D. How to take care of the psychological health of patients with neurogenic bladder?

Theme 3: Evaluation and Follow-Up of Upper and Lower Urinary System Function in Patients With Neurogenic Bladder

A. How do you monitor and evaluate the upper urinary tract system, and how long does it take?

B. How do you monitor and evaluate the lower urinary tract system, and how long does it take?

Theme 4: Urinary Tract Infections Associated With Neurogenic Bladder Disease and How to Deal With It

A. How long does it take to repeat the urine culture examination in patients with neurogenic bladder?

B. If the patient has asymptomatic bacteriuria, do you prefer treating him with antibiotics - taking into account the presence of no catheters/intermittent catheters/indwelling catheters? If you decide to treat him with antibiotics, what types of antibiotics do you usually choose? How much treatment period do you decide?

C. If the patient suffers from symptomatic UTI, what types of antibiotics do you usually choose for treatment? How much treatment period do you decide?

D. Do you support the use of antibiotics to prevent recurrent urinary tract infections? What do you think of the self-use of antibiotics or prevention with a daily dose?

E. Do you have a standard protocol for the treatment of recurrent urinary tract infections in patients with neurogenic bladder?

Theme 5: Opinions and Future Attitudes in the Treatment of Neurogenic Bladder Patients

A. Do you think that patients with neurogenic bladder receive appropriate care and treatment in the place or institution in which you work? Or do they need to be transferred to more specialized centers?

B. What are the future recommendations based on your scientific and practical experience that you suggest in the follow-up and treatment of neurogenic bladder disease?

Theme 6: Neurogenic Bladder in Patients With Multiple Sclerosis

A. Are additional tests performed to exclude other diseases that cause nervous bladder, such as multiple sclerosis?

B. How many patients are diagnosed with neurogenic bladder, and then it turns out that there is a disease causing this condition, such as multiple sclerosis? Are there patients diagnosed with multiple sclerosis to be referred to you for diagnosis and follow-up of neurogenic bladder symptoms?

C. Are there unified protocols for dealing with patients with multiple sclerosis who suffer from neurogenic bladder?
